# Rhodamine 6G-Ligand Influencing G-Quadruplex Stability and Topology

**DOI:** 10.3390/ijms22147639

**Published:** 2021-07-16

**Authors:** Lukáš Trizna, Ladislav Janovec, Andrea Halaganová, Viktor Víglaský

**Affiliations:** 1 Department of Biochemistry, Institute of Chemistry, Faculty of Sciences, Pavol Jozef Šafárik University, 04001 Košice, Slovakia; lukas.trizna@yahoo.com (L.T.); andreahalaganova@gmail.com (A.H.); 2 Department of Organic Chemistry, Institute of Chemistry, Faculty of Sciences, Pavol Jozef Šafárik University, 04001 Košice, Slovakia; ladislav.janovec@upjs.sk

**Keywords:** G-quadruplex, ligand, rhodamine, thiazole orange, thioflavin T

## Abstract

The involvement of G-quadruplex (G4) structures in nucleic acids in various molecular processes in cells such as replication, gene-pausing, the expression of crucial cancer-related genes and DNA damage repair is well known. The compounds targeting G4 usually bind directly to the G4 structure, but some ligands can also facilitate the G4 folding of unfolded G-rich sequences and stabilize them even without the presence of monovalent ions such as sodium or potassium. Interestingly, some G4-ligand complexes can show a clear induced CD signal, a feature which is indirect proof of the ligand interaction. Based on the dichroic spectral profile it is not only possible to confirm the presence of a G4 structure but also to determine its topology. In this study we examine the potential of the commercially available Rhodamine 6G (RhG) as a G4 ligand. RhG tends to convert antiparallel G4 structures to parallel forms in a manner similar to that of Thiazole Orange. Our results confirm the very high selectivity of this ligand to the G4 structure. Moreover, the parallel topology of G4 can be verified unambiguously based on the specific induced CD profile of the G4-RhG complex. This feature has been verified on more than 50 different DNA sequences forming various non-canonical structural motifs.

## 1. Introduction

G-quadruplexes (G4s) are relatively common in the genomes of all living cells, including viruses, but their frequency differs from species to species [1,2]. The G4 motif may be an integral part of some artificially developed DNA and RNA aptamers [3,4,5,6]. The dispersion of putative G4 sequences in genomes is not random, however, and their localization is closely correlated with specific gene functions [7]. An investigation of different genomes using various searching algorithms indicated that at least 3.10^5^ and up to 3.10^6^ G4-putative sequences can be formed in the human genome [8,9]. Therefore, in the past decade, considerable efforts have been made with the aim of developing small molecular probes capable of selectively recognizing G4s in therapeutic drug screening and biosensor construction since DNA molecules are not readily visible in such assays [10,11,12,13,14]. 

An extremely wide range of fluorophores which target nucleic acids have been identified to date, and several of these optical probes are routinely used in fluorescent microscopy studies to stain genetic material in the nucleus (e.g., DAPI and Hoechst) [15,16]. Frequently, π-π interactions between polyaromatic systems and nucleobases play a crucial role in determining the binding mode, typically through intercalation and insertion in between base pairs of duplex DNA or in end-stacking on the G-quartets of G4s. In addition to these binding modes, ligands can also bind to the grooves of DNA or through direct coordination. Additionally, electrostatic interactions play an important role in increasing the affinity between positively charged optical probes and the negatively charged phosphates found in nucleic acids.

**Figure 1:**
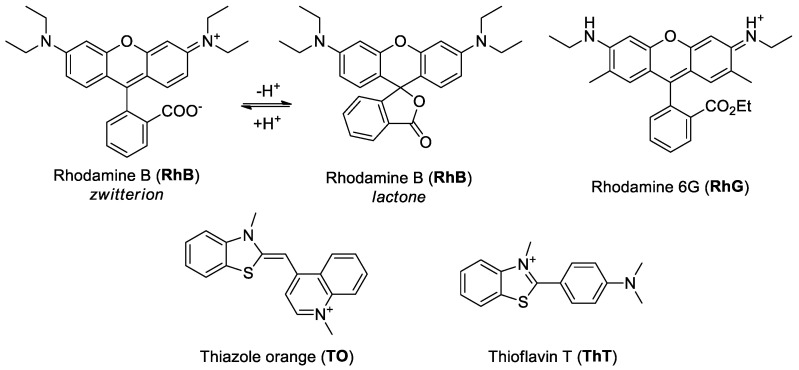
Structure of ligands directly used in this study.

In this study we investigate the interaction of series of known G4-DNA forming oligonucleotides with rhodamine dyes containing a fluorescent xanthene core; specifically, Rhodamine B (RhB) and Rhodamine 6G (RhG). The results were compared with those obtained for other well-characterized G4 ligands, primarily Thiazole Orange (TO) and Thioflavin T (ThT), Figure 1.

RhB is a widely available dye which is commonly used as a water tracer or as a colorant in textiles and foodstuffs, but it can also serve as a fluorescent biomarker [17,18]. Nanoparticles consisting of RhB derivatives have shown considerable potential for applications in the field of biomedical sciences [19]. RhG is an organic laser dye which is suitable for use in studying the probes as it has a high quantum yield for fluorescence. As with RhB, this agent has a wide range of potential applications, ranging from use as a fluorescence tracker which can help in defining the spectroscopic characteristics with a high conversion efficiency and precision to its use as a leukocyte marker [20]. No cytotoxicity has been detected for RhG at µM concentrations in vivo [21]. ThT is an effective fluorescence probe in the detection of DNA and RNA G4s. Nucleolar G-quadruplexes in living cells have been visualized by using ThT and the high selectivity of the ligand allows researchers to distinguish between G4 and non-G4 structures [22]. The cyanine dye TO is widely used as a fluorescent probe which becomes illuminated upon binding to almost all forms of DNA, but the dye exhibits poor selectivity in differentiating G-DNA from other structural forms of DNA [23].

The adopted structure of each oligonucleotide was verified using UV absorption, CD spectroscopy and electrophoretic separation in the presence of either sodium or potassium ions. Circular dichroism (CD) spectroscopy has been used to monitor spectral profiles of non-canonical structural motifs structure formation under different conditions, mainly the presence of ligands and cations. This method was also combined with other techniques to identify other properties of the folded structures such as multimerization and stability. For this purpose, various types of electrophoreses, UV-Vis absorption and fluorescent spectroscopies were performed. Parallel and antiparallel G4 topologies can typically be identified by determining the position of the positive and negative peaks in CD spectra in the UV range of 230−320 nm [24]. In order to eliminate the false confirmation of conformation on the basis of CD spectra profiles alone, CD melting curves and temperature gradient-gel electrophoresis (TGGE) were used because, as is generally known, the stability and melting temperature of G4s are significantly higher in the presence of potassium than in the presence of sodium ions [25]. Other non-canonical forms are significantly less sensitive to the presence of potassium. In addition, the ligand gradient-gel electrophoresis (LGGE) was also used in this study to monitor the influence of ligand to G4 topology. 

The main goal of this study is to demonstrate that the fluorophore RhG selectively binds to parallel forms of G4s. In order to verify the relatively high selectivity, other sequences capable of forming non-canonical structures were also analyzed.

## 2. Results and Discussion

### 2.1. The Spectral Properties of DNA-Ligand Complexes

Parallel G-quadruplex structures exhibit a clear positive band at ~265 nm and a negative peak at ~240 nm, while antiparallel G4 structures exhibit a positive CD signal at ~295 nm and a negative signal at ~265 nm. In contrast, the so-called (3 + 1) conformer, in which three strands are in the same alignment with another strand oriented in the opposite direction, exhibits a positive shoulder at 265−270 nm, but it should be noted that a mix of parallel and antiparallel structures can show signatures which are close to the topology of (3 + 1) conformers [26,27]. However, other structural forms may display a positive peak close to 265 nm, but this spectrum does not necessarily indicate the presence of a G-quadruplex [28,29]. CD spectra can also be used for the detection of i-motifs; the maximum and minimum Cotton effects at 288 and 258 nm are indicative of the formation of this structure and the peaks at 275 and 249 nm are indicative of unstructured DNA [30]. Interestingly, some achiral ligands binding to DNA form a chiral complex which shows an induced CD (ICD) signal close to the wavelength region of absorption, but it should be noted here that ICD can also be observed in the UV region in which G4s show a characteristic CD signal with the UV-ICD signal interfering with an original signal corresponding to G4 formation. The results show the unique ICD profile in visible region caused by G4-ligand interaction [31,32]. A ligand causing ICD in a G4-ligand complex usually stabilizes the G4 motif, but it can also induce topological changes and facilitate G4 multimerization [27]. On this basis, ICD signatures can be used to determine whether a sequence forms G4 motif. 

More than 50 different oligomeric sequences which form different non-canonical structures have been analyzed; see the Material and Methods section for more details. 

**Figure 2:**
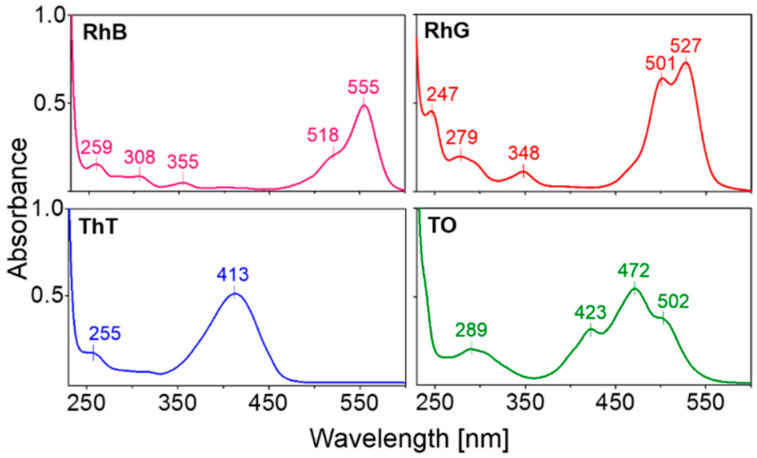
Absorption spectra of 130 µM ligands used in this study in a 25 mM mRB buffer, pH 7.0.

The UV-Vis spectra of the studied ligands are shown in Figure 2. In the absence of DNA, each ligand shows an absorption signal in the visible wavelength in the range of 350–560 nm, and an ICD signal is therefore expected in this region.

**Figure 3:**
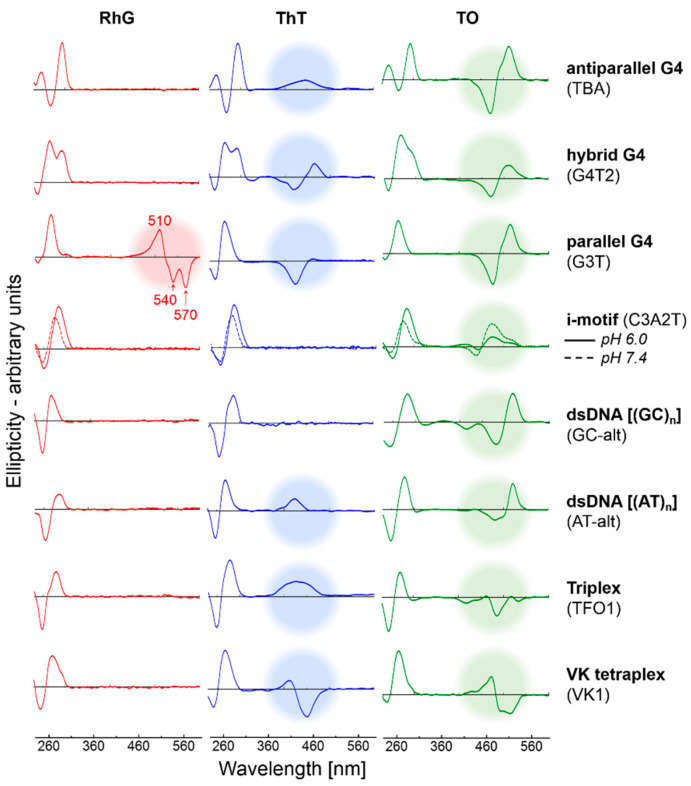
Representative CD spectra of DNA sequences able to adopt different non-canonical structures. The concentration of DNA and ligand was 27 and 130 µM (~1:5 eqv.), respectively, in each measurement. ICD signals are highlighted with colored circles. The mBR contains 50 mM NaCl. The positive and two negative peaks are observed at ~510, ~540 and ~570 nm, respectively.

The representative spectral results for various non-canonical motifs in the presence of various ligands are shown in Figure 3. All DNA sequences summarized in the Table 1 were analyzed using CD and absorption spectroscopy. 

The results clearly demonstrate that, with the exception of RhB, ICD signals only occur when G4s are formed regardless of the ligand used. However, the results also suggest the poor selectivity of ThT and TO, with ICDs being observed for many different non-canonical forms, including that of the dsDNA-ThT complex; the presence of ICD is a signature of DNA-ligand interaction. Nevertheless, the profile of the G4-TO complexes shows some common features as has been demonstrated in our previous study [31,32]. The profile of G4-TO complexes is very similar to those of other G4 putative sequences. However, the ICD signal obtained with TO does not allow us to distinguish between parallel and antiparallel G4 topologies. In contrast, the ICD signal with RhG is observed only in the case of parallel G4 topologies, and we can therefore suggest that the selectivity of RhG is restricted for the determination of parallel G4s. In addition, the presence of salts was found to have interfered only slightly with the shape of ICD profiles, Figure 3. Any parallel G4 structure exhibits almost the same CD spectral features as those obtained with the G3T oligonucleotide in the presence of RhG.

#### RhG versus RhB

In contrast to RhB, RhG is positively charged in a neutral condition, and a significantly higher affinity with DNA would therefore be expected. In addition to its above-mentioned affinity G4s, its significant advantage of RhG over RhB is the presence of an ethyl ester group in its structure, Figure 1. The presence of an ester protects the carboxyl group and blocks the formation of a lactone cyclic structure, resulting in the greater stability of the RhG structure. RhB is also able to form cyclic forms, a factor which increases its structural variability, and which may also explain why the affinity for G4 structures is not as pronounced as that recorded for RhG. In addition, the carboxyl group forms a COO-anionic structural form under certain environmental conditions (e.g., high pH), which also has an adverse effect on its affinity for negatively charged DNA [33].

### 2.2. RhG: Influence on Polymorphism and Stability 

**Figure 4:**
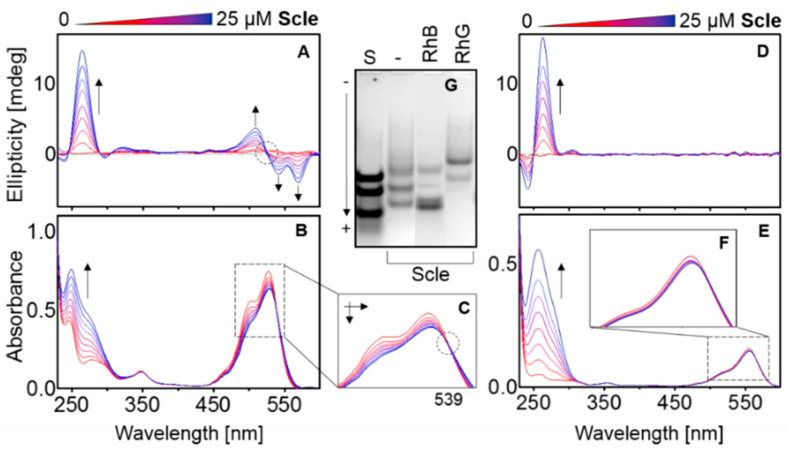
CD and UV-Vis spectra of 130 µM RhG (**A**,**B**) and RhB (**D**,**E**) in mRB, supplemented with 50 mM KCl, pH 7.0. The increment of the Scle oligomer is 3.35 µM. The final concentration of DNA is ~27 µM (0.2 ekv). The enlarged UV-Vis region of the RhG absorption spectra shows a clear isosbestic point at 539 nm (**C**), but not in (**F**). 12% PAGE (**G**) in corresponding conditions; the concentration of the ligand in the two columns is 130 µM. The standard is a mixture of oligonucleotides AC9, AC18 and AC28.

The Scle core sequence (d[TGGGGGGGTGGGTGGGT]) derived from the sclerostin binding aptamer [34] adopts a clear parallel G4 structure in the presence of 50 mM potassium; a positive CD signal is observed at 265 nm, Figure 4. The results also show an influence of increasing concentration of DNA in the presence of 130 µM of RhB and RhG. The RhG ICD signal strength is dependent on Scle concentration. The electrophoretic separation shows unambiguously that at least three different folds of Scle can be formed under the given conditions. A clear isosbestic point at ~539 nm in the absorption spectra was also observed in the RhG spectra, panel C. However, the RhB spectra indicate a different pattern of behavior; a negligible effect on spectral shift and an unclear isosbestic point were detected, panel F. Nevertheless, the influence of both RhB and RhG on the distribution of topological forms are also clear from the results; the intensity and position of bands are different from those observed in the PAGE experiment without the presence of the ligands. An intensive ICD signal was also observed for the Scle-RhG complex. G3T showed a very similar sequence to that of Scle is G3T, with only one G base being substituted by T.

**Figure 5:**
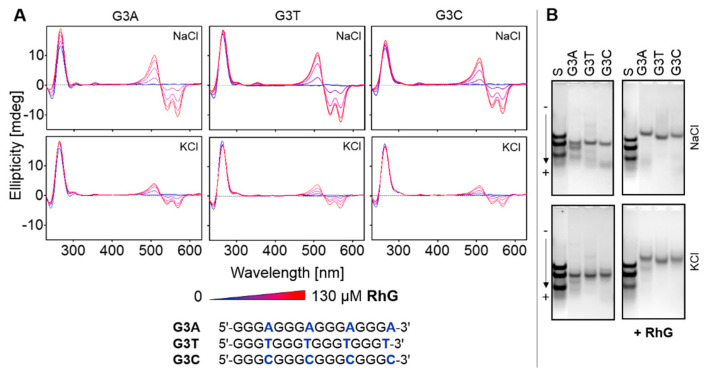
CD titration spectral measurements and PAGE of d(G_3_NG_3_)G_3_ sequences (~27 µM) at different ionic conditions in the presence of increasing concentrations of RhG up to 260 µM (**A**). The increment of RhG is ~33 µM. The left and right PAGE panels (**B**) represent electrophoretic records in the absence and presence of 130 µM of ligand, respectively. Electrophoresis was performed in the presence of both 50 mM NaCl and KCl. The mixture of AC9, AC18 and AC28 is used as standard.

The results shown in Figure 5 demonstrate the effect of RhG on a series of sequences d(G_3_NG_3_)G_3_, where N represents either C, T or A, respectively. Any of these sequences adopts parallel G4 in either the presence or absence of RhG ligand regardless of whether sodium or potassium cations are present; CD positive peaks are observed at 265 nm. CD spectral features for this set of oligonucleotides are almost identical to those observed for the core of Scle sequence. The electrophoretic results show the effect of RhG on electrophoretic mobility and on the elimination of the number of folds, primarily for the less stable G3A oligomer which shows the highest level of polymorphism. These set of oligonucleotides preferentially form dimeric structure. These results suggest that RhG has a significant effect on the folding process, with multimeric topological forms being facilitated. The electrophoreses of other sequences adopting different non-canonical motifs are summarized in Supplementary Figure S1. These results also confirm the effect of ligand-induced multimerization. 

### 2.3. Temperature and Concentration Measurements

CD and UV melting analyses were performed using the method described in our previous studies [27,28]. However, as has already been mentioned, DNA sequences rarely adopt only a single well defined and stable conformation, and instead typically form a wide range of different topological isoforms. This feature may explain why the spectral measurements display a melting curve which represents the average melting of a mix of topological forms which have occurred in the solution. It is therefore not appropriate to apply van’t Hoff analysis in the case of this type of melting curve as this approach is intended for use with two-state systems [35,36]. As a result, it should be noted that the selected values of melting temperature of G4s and one triplex and i-motif obtained using CD spectroscopy which are summarized in Table 1 represent only indicative values. In addition, spectral melting curves of this type cannot offer an unambiguous explanation of declination from the two-state mechanism. The corresponding electrophoreses are shown in Supplementary Figure S1.

**Figure 6:**
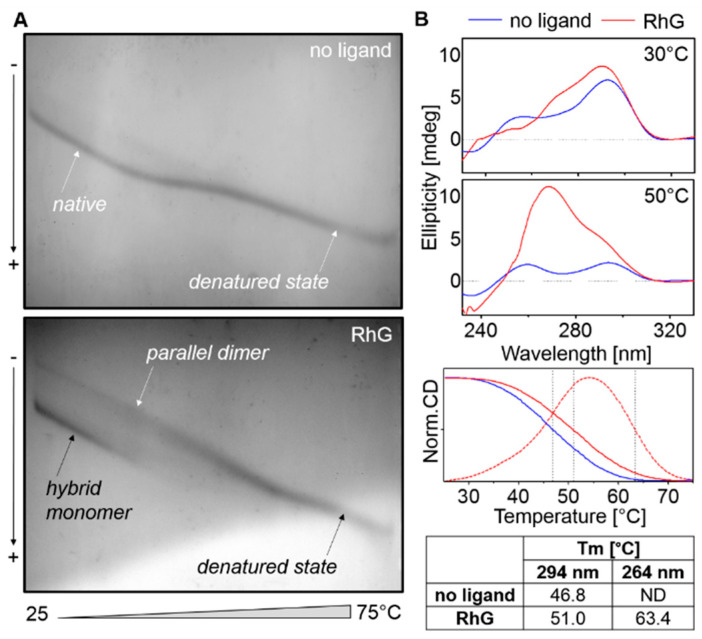
(**A**) TGGE record of HTR sequence in 25 mM mBR, pH 7.0 supplemented with 2.5 mM KCl (up). The corresponding electrophoretic result contained 260 µM of Rh6G (down). (**B**) CD spectra under the same conditions as the TGGE assay in the presence and absence of RhG. The temperature dependences were obtained at 264 (red dashed line) and 294 (solid lines) nm. CD melting temperatures are shown in the enclosed table. These temperatures agree with those obtained with TGGE: 46.5 °C and 50.6 °C for antiparallel G4 in the absence and presence of RhG, respectively, and >62 ± 2 °C for parallel G4 with RhG.

Although the values are only indicative, it is clear that antiparallel and hybrid G4 conformers are only slightly stabilized with RhG, but the melting temperature of parallel G4s shows a more significant increase. The triplexes and i-motifs have been destabilized with RhG. In order to provide clearer evidence, G4 forming sequences were also examined using TGGE. The results shown in Figure 6 include TGGE results for HTR in both the presence and absence of RhG in the gel. In order to eliminate the occurrence of a high electric current only 2.5 mM of KCl was used. The corresponding CD measurements under identical conditions may help to clarify the melting mechanism and the influence of RhG on this process. Parallel G4 structures were not found to be the dominant form at lower temperatures, even with the presence of RhG, but a temperature increase resulted in an increasing population of parallel G4 structures at the expense of antiparallel hybrid conformations. This change can be seen clearly in the dotted CD melting curve obtained at 264 nm. However, the parallel topology was also found to be more stable than the hybrid structure.

**Figure 7:**
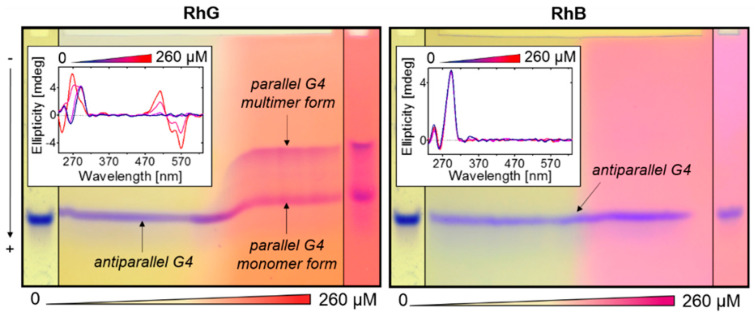
LGGE of HIV-M27 (d-[GTGGCCTGGGCGGGACTTGGGA]) performed in a 25 mM mRB buffer, pH 7.0 supplemented with 50 mM KCl and 0 to 260 µM rhodamines RhG (**left**) and RhB **right**). The concentration of polyacrylamide was 12%. The inset represents a corresponding CD spectrum under the same conditions. The concentration of ligand in CD was 0–260 µM, the increment is 65 µM. The G4 conversion from antiparallel to parallel monomer and dimer is highlighted with arrows. The left and right columns represent standard PAGE of HIV-M27 performed in gels containing 0 and 260 µM of ligands, respectively.

The TGGE profiles show clearly that the mobility of the parallel G4 dimer differs only slightly from that of the denatured form, and therefore this method is not generally applicable in melting analyses of any G4 structures. Another interesting example which demonstrates the influence of the ligand to G4 structure is the unorthodox arrangement found in the LGGE electrophoresis, in which the nonlinear gradient of the ligand is applied in a perpendicular direction to the sample movement, Figure 7. This methodology allows some details concerning the interaction of DNA with the ligand and the folding process to be clarified. The results demonstrate the influence of the studied rhodamines on the multimerization of G4 and on the occurrence of other topologies. Increasing concentrations of RhG resulted in an abundance of parallel G4 structures but also of multimeric forms. The isothermal analysis performed at laboratory temperature enables the visualization of the occurrence of native conformers at various concentrations of the ligand. A sequence which occurs in the HIV genome was used for this purpose [37]. The effect observed in the case of RhG was not recorded with RhB, a result which is probably due to the weak interaction of the ligand with G4. No conversion to parallel topology or multimerization occurred and, of course, no ICD signal was detected, not even in the presence of other oligonucleotides. Nonetheless, RhB did exert a slight stabilization effect on G4 structures, an effect which was primarily observed in conditions in which sodium was present, but potassium was absent; the T_m_ value was seen to have increased by approximately 0.5–3 °C (not shown). The continuous mobility profile of the electrophoretic band allowed the entire topological conversion connected with the multimerization of the appropriate sequence to be monitored. Using a combination of LGGE with, for example, CD titrating analysis, it is possible to determine many of the “hidden” details of ligand-DNA interaction. This methodology is introduced here for the first time, and we believe that it will prove to be a useful tool in future DNA-ligand interaction studies.

### 2.4. Fluorescence Spectroscopic Properties of RhB and RhG

**Figure 8:**
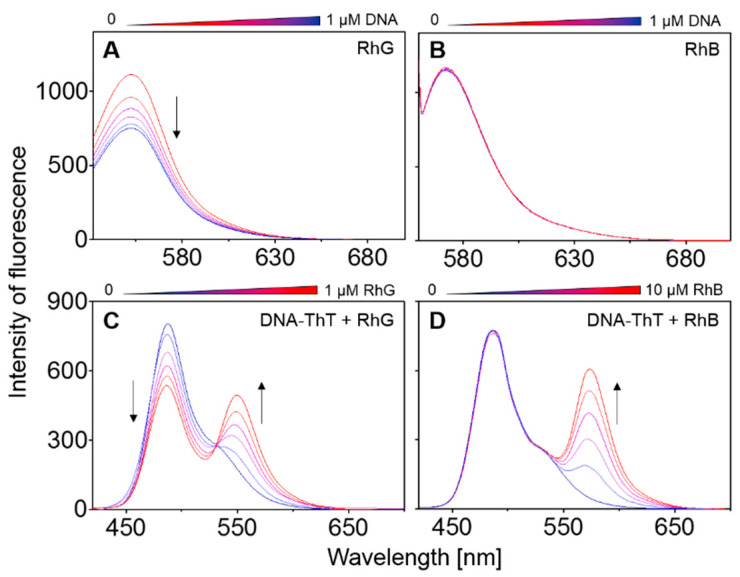
Fluorescence titration of 25 nM RhG (**A**) and RhB (**B**) with the stepwise addition of the G3T oligonucleotide (0, 0.2, 0.4, 0.6, 0.8 and 1.0 µM) corresponding to 0−40 molar equivalents. Increasing concentrations of DNA are highlighted with arrows. Measurements were performed in a mRB buffer supplemented with 50 mM KCl at a pH of 7.0; excitation of RhG and RhB at 527 nm and 555 nM, respectively, the excitation and emission slits were 2.5 nm (5 nm in B) and the scan speed was 240 nm/min. 1 µM G4-ThT mixture (1: 1 molar eqv.) titrated with 0–1 µM RhG (**C**) and 0–10 µM RhB (**D**), the excitation was at 413 nm.

The two reference fluorescent ligands ThT and TO which target G4 structures have been studied in depth as fluorescent G4 ligands, with both dyes displaying considerable fluorescence yields when bound to G4 in comparison to their independent fluorescence in solution without the target structures [38,39]. The selectivity of TO was lower than that of ThT, and TO also displays a strong illumination effect upon association with various topological forms of nucleic acids [38]. The binding constants of the two agents are in the micromolar range [40,41]. As was mentioned above in the introduction, rhodamines are also fluorophores, and it is therefore appropriate to analyze their interactions with DNA using fluorescence spectroscopy, thereby allowing the DNA-rhodamine complexes to be determined in more detail. The most relevant results are shown in Figure 8. The fluorescence of RhG was quenched when the ligand interacted with DNA, but the strongest effect was observed for parallel G4 structures, panel A, although other structural forms of DNA were found to quench RhG fluorescence to a less significant degree (not shown). However, RhB quenching was almost negligible for all the DNA sequences used, including those featuring G4 motifs, panel B. As evidence of the affinity of RhG, the promising G4 ligand of ThT was displaced from the ThT-G4 complex by RhG and RhB, panel C and D, respectively. It is evident that the signal corresponding to the ThT-G4 complex at ~485 nm was eliminated at increasing amounts of RhG and the signal at 555 nm corresponding to RhG was found to increase; ThT is displaced by RhG. However, this effect was not observed in the case of RhB. A crossover point analogical to the isosbestic point was also observed which indicates that these spectra are coupled. Even though the concentration of RhB was 10-fold higher, no light-down corresponding to ThT-G4 complex was observed. Although the determination of the binding constant of RhG to DNA was not a primary aim of this study, it was possible to estimate this value based on the results of the experiments monitoring G4 ligand displacement by rhodamines. The binding constant of RhG falls in the same region as that of ThT because an equivalent amount of RhG can displace ThT. However, this constant varies, and it is dependent on the G4 topology and the presence of cationic molecules. Due to the complexity of this relationship, a deeper analysis of the binding constant lies beyond the scope of this study. 

### 2.5. Molecular Modeling of Ligand-G4 Interactions

**Figure 9:**
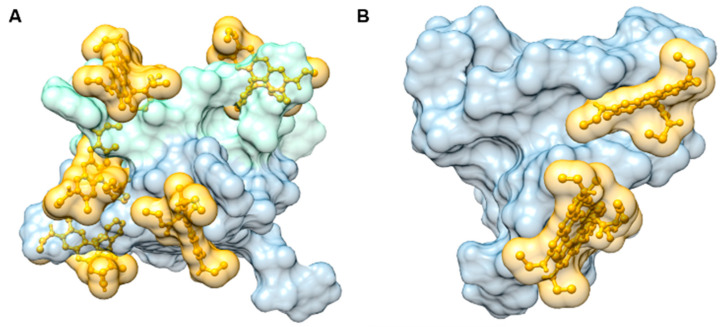
Putative binding of the RhG ligand within the quadruplex structure PDB 2le6 (**A**) and 2jpz (**B**) obtained from docking simulations. Only the leading structures of the most populated binding clusters are depicted. The quadruplex is drawn in a solvent-accessible surface representation. The ligand is shown in a ball and stick representation. The solvent-accessible surface of the ligand is also shown. The ligand is shown fitting into the quadruplex grooves. The subunits of 2le6 are colored with different hues, pale green and blue grey. Images were prepared using Chimera software [43].

Docking simulations were also carried out to demonstrate the putative binding of RhG within G4 structures. The simulations of ligand binding were performed with structures representing parallel dimer (2le6) and hybrid (2jpz) G4 structures because RhG can be shown to affect these topologies, Figure 9. 2le6 [5′-d(GIGTGGGTGGGTGGGT)-3′] and 2jpz [5′-d(TTAGGGTTAGGGTTAGGGTTAGGGTT)-3′] represent structural analogs to G3T and HTR structures, respectively. The docking simulation may not represent the true structure of the DNA-ligand complex because the ligand interaction may slightly alter the initial coordinate values of the atoms in the G4 structure, and this declination may continue until the complex reaches its most stable form. The docking simulation identifies the best configuration for a fixed structure in terms of the given data. Nevertheless, the 2le6 structure could represent a structure which is close to that induced with RhG. The most populated binding clusters of the five anchored RhGs could be a source of the strong ICD signals, e.g. Figure 4a. The best matches were obtained for structures in which RhG was bound into the G4 grooves close to cavity formed by G4 loop. Although this type of molecular modeling did not confirm the presence of stacking interactions with terminal G-quartets, an arrangement which is typically observed with NMR or crystallographic data, we cannot rule out the possibility that such an interaction occurs in G4-RhG complexes [41,42]. Nevertheless, this set of results demonstrates possible places where the initial attachments of RhG with the folded G4 structure occur and not a consequent G4 structure modification driven by the ligand. We realize that PDB sequences are not the same as studied oligomers, but results obtained with docking simulation show that G4 structures contain the binding sites tailored for RhG.

## 3. Materials and Methods

All chemicals and reagents were obtained from commercial sources. DNA oligonucleotides were obtained from Metabion, Germany (Table 2). PAGE purified DNA was dissolved in double distilled water prior to use. Thiazole Orange, Thioflavin T, Rhodamine B and Rhodamine 6G were purchased form Merk, Slovakia (390062, T3516, R6626, 252441). Single strand concentrations were determined precisely by measuring absorbance (~260 nm) at 95 °C using molar extinction coefficients [44]. DNA concentrations were determined using UV measurements carried out on a Jasco J-810 spectropolarimeter (Easton, MD, USA).

### 3.1. Circular Dichroism Spectroscopy

CD spectra were recorded on a Jasco J-810 spectropolarimeter equipped with a PTC-423L temperature controller using a quartz cell of 1 mm optical path length in a reaction volume of ~150 µL; instrument scanning speed of 100 nm/min, 1 nm pitch and 1 nm bandwidth, with a response time of 2 s. CD data represents three averaged scans taken at a temperature range of 0–100 °C. Scans were performed over a range of 220–700 nm. All other parameters and conditions were the same as those which were described previously [27]. A modified Britton–Robinson buffer (mBR) in which TRIS was used instead of potassium/sodium hydroxide (25 mM phosphoric acid, 25 mM boric acid and 25mM acetic acid) was used in all spectral analyses and was supplemented by either 2.5–50 mM potassium chloride or 50 mM sodium chloride; pH was adjusted by TRIS to a final value of 7.0. I-motif was also measured in acidic conditions (pH 4–6). DNA titration was performed with increasing concentrations of the ligand. Each ligand was solubilized in DMSO or ethanol to reach a final concentration of 10 mM in the stock solution. The concentrations of DNA and ligand in the 1 mm quartz cell were 30 µM and 0–200 µM, respectively, and the increment of the ligand was ~67 µM. Each sample was mixed vigorously for 3 min following the addition of ligand; CD/UV spectra were performed immediately.

### 3.2. CD Melting Curves

CD melting profiles were collected at ~295 and ~265 nm as a function of temperature using a procedure which has been published previously [27,36]. The temperature ranged from 0 to 100 °C, and the heating rate was 0.25 °C per minute. The melting temperature (Tm) was defined as the temperature of the mid-transition point.

### 3.3. Electrophoresis 

Samples consisting of 0.3 µL of 1mM stock solutions were separated using nondenaturing PAGE in a temperature-controlled electrophoretic apparatus (Z375039-1EA; Sigma-Aldrich, San Francisco, CA, USA) on 12% acrylamide (19: 1 acrylamide/bisacrylamide) gels. DNA was loaded onto 13 by 16 by 0.1 cm gels. Electrophoresis was run at 10 °C for 2 h at 125V (~8 V⋅cm^−1^). Each gel was stained with StainsAll (Sigma-Aldrich). All electrophoretic measurements were performed in a mBR buffer at pH 7.0. Temperature gradient gel electrophoresis (TGGE) equipment was used according to a method which has been described previously [44,45]. The gel concentration was 12%. Electrophoreses were run perpendicularly to the temperature-gradient (20–80 °C) for 3 h at 160 V (~8 V·cm^−1^). Approximately 12 μg of DNA was loaded into the electrophoretic well. DNA oligomers were visualized with Stains-all after the electrophoresis. Ligand gradient gel electrophoresis (LGGE) is similar to denaturing gradient gel electrophoresis (DGGE), but in place of a denaturing agent, a concentration gradient of ligand (0–260 µM) is applied perpendicularly to the movement of the DNA sample. The same apparatus used for standard PAGE analyses was used in this assay. The technique was developed and applied in our laboratory to monitor the folding and multimerization effect of the ligands on G4 structures.

### 3.4. Fluorescence Spectroscopy

Fluorescence spectra were acquired at 20 ± 1 °C with a Jasco FP-8300 Spectrofluorometer which was equipped with a Peltier temperature controller ETC-815. A quartz cuvette with a 10 mm path length was used in all experiments. In the fluorescence measurements, the excitation and emission slits were 2.5–5 nm, and the scan speed was 240 nm/min. Then, 25 nM of ligand was titrated with DNA (0–1 µM) in a mRB buffer in both the presence and absence of monovalent metal cations. The molar ratios between DNA and ligand were 1:40, 1:32, 1:24, 1:16 and 1:8. The excitation wavelength was adjusted to 527 and 413 nm for RhG and ThT, respectively.

### 3.5. Docking Studies

Molecular models of RhG was created using the building options in an ACD/ChemSketch (ACD/ChemSketch package 2020.2.0 www.acdlabs.com (accessed on 17 June 2021). The models were built as 3D structures and saved as *Mopac* input files using the ACD/3D Viewer (ACD/3D Viewer package 2020.2.0 www.acdlabs.com (accessed on 17 June 2021). MOPAC2016 was used to optimize the ligand geometry [46]. Chimera software [43] was used to extract coordinates of a G4 structure from a pdb file id: *2jpz* [47]; *2le6* [48]. As NMR spectroscopy was used to determine the coordinates of a G4 structure, only the coordinates of the first model were selected for a docking simulation. In the case of the quadruplex id: *2le6*, the position of the last residue dT(16) was changed in order to allow stacking interactions with a ligand during the docking run. The position of the last nucleotide was changed using the Structure Measurements module in the Chimera software program; the torsion angle defined as dG(15).A C3′–dG(15).A O3′–dT(16) P–dT(16) O5′ was changed from −74 to +74 degrees. MGL TOOLS 1.5.6 software was used to assign Gasteiger partial atomic charges to the G4 structure [49]. The Antachamber module of the Ambertools 18 software package was used to derive charges for the ligands via the AM1-bcc method. Docking simulations were carried out using Autodock ver. 4.2, while MGL TOOLS 1.5.6 was used to prepare the input files [50]. United atom representations were used for the ligands and G4 structures. The grid for energy for G4s pdb id: *2jpz* and *2le6* was set at the center of the macromolecule with the dimensions of 120 × 120 × 120 points (x,y,z) and a spacing of 0.375 Ǻ. Docking runs were performed using a Larmarckian genetic algorithm. Docking began with a population of random ligand conformations in a random orientation and at a random translation. Each docking experiment was derived from 100 different runs which were set to terminate after a maximum of 25 × 10^5^ energy evaluations or 27 × 10^3^ generations, yielding 100 docked conformations. The population size was set to 300. For other parameters, the default values were used. Five docking runs were performed for the ligand.

## 4. Conclusions

In conclusion, the results of the study indicate that RhG acts as a promising stabilizer of G4 structures. The ligand was found to bind preferentially to parallel G4 topologies and to promote G4 multimerization, while the fluorescence quenching induced with G4, and the resultant ICD values are highly significant in comparison with other DNA structures. The findings of the computer modeling predict that RhG binds to the grooves of G4 structures. In addition, LGGE is the first application to our knowledge that can demonstrate the concentration effect of the ligand on the G4 topology. Our results regarding the selectivity of RhG to G4s could serve as a starting point for the development and synthesis of novel fluorescent organic and metalloorganic G4-probes derived from the basic skeleton of RhG. Highly selective optical probes are frequently required for the construction of functionalized-nanoparticles and drug delivery systems, and therefore these types of small molecules show great potential for future applications in molecular biology and in a wide range of biomedical fields.

## Figures and Tables

**Table 1:** Selected melting temperatures obtained using CD spectroscopy.

		Melting Temperature [°C]			
Oligo	Wavelength [nm]	50 mM NaCl		50 mM KCl	
		No Ligand	RhG	No Ligand	RhG
HTR	294	52.0	52.0	63.5	64.0
Scle	264	62.0	77.0	81.5	>100
TBA	294	20.0	26.8	46.5	48.5
Hema	264	ND	54.3	72.0	86.0
STAT	264	54.5	76.5	92.8	>100
HCV	264	44.5	60.0	72.6	86.0
ionK	294	46.2	48.5	59.1	58.0
VEGF	264	47.5	82.0	85.6	87.9
		no ligand ^a^		RhG ^a^	
C3A2T ^b^	286	28.3		24.3	
TFO1	282	20.4		17.7	

^a^ obtained in absence of salt, ^b^ obtained in pH 6.0 and ND—not determined.

**Table 2:** Sequences of oligonucleotides used in the present study.

No.	Name	Sequence in 5′→3′ Direction	Category and Preferred Motif	
1	**G3A**	GGGAGGGAGGGAGGGA	**G_3_N_n_** [31]	**G-quadruplex**
2	**G3C**	GGGCGGGCGGGCGGGC		
3	**G3T**	GGGTGGGTGGGTGGGT		
4	**G3T2**	GGGTTGGGTTGGGTTGGG		
5	**G3T3**	GGGTTTGGGTTTGGGTTTGGG		
6	**G3T4**	GGGTTTTGGGTTTTGGGTTTTGGG		
7	**HTR**	GGGTTAGGGTTAGGGTTAGGG		
8	**HTR2**	AGGGTTAGGGTTAGGGTTAGGGT		
9	**HTR-T**	GGGTTAGGGTTAGGGTTAGGGT		
10	**G3T2C**	GGGTTCGGGTTCGGGTTCGGG		
11	**8G3**	GGGTTAGGGTTAGGGTTAGGGTTAGGGTTAGGGTTAGGGTTAGGG		
12	**8G3T2**	GGGTTGGGTTGGGTTGGGTTGGGTTGGGTTGGGTTGGG		
13	**8G3T3**	GGGTTTGGGTTTGGGTTTGGGTTTGGGTTTGGGTTTGGGTTTGGG		
14	**G3-3-A20**	GGGTTAGGGTTAGGGTTAGGGAAAAAAAAAAAAAAAAAAAA		
15	**G3-5-T20**	TTTTTTTTTTTTTTTTTTTTGGGTTAGGGTTAGGGTTAGGG		
16	**G4T**	GGGGTGGGGTGGGGTGGGG	**G_4_N_n_** [31]	
17	**G4T2**	GGGGTTGGGGTTGGGGTTGGGG		
18	**G4T3**	GGGGTTTGGGGTTTGGGGTTTGGGG		
19	**G4T4**	GGGGTTTTGGGGTTTTGGGGTTTTGGGG		
20	**G4T2A**	GGGGTTAGGGGTTAGGGGTTAGGGG		
21	**HCV**	GGGCGTGGTGGGTGGGGT	**Aptamers** [45]	
22	**Hema**	GGGGTCGGGCGGGCCGGGTG		
23	**HIV**	GGGGTGGGAGGAGGGT		
24	**Insu**	GGTGGTGGGGGGGGTTGGTAGGGT		
25	**ionK**	GGGTTAGGGTTAGGGTAGGG		
26	**OCH-A**	CGGGTGTGGGTGGCGTAAAGGGA		
27	**Scle**	TGGGGGGGTGGGTGGGT		
28	**STAT**	GGGCGGGCGGGCGGG		
29	**TBA**	GGTTGGTGTGGTTGG		
30	**TBA-5T**	GGTTGGTGTGGTTGGTTTTTGGTTGGTGTGGTTGG		
31	**VEGF**	GGGGCGGGCCGGGGGCGGG		
32	**HIV1-K02**	GTGGCCTGGGCGGGACTGGGGA	**HIV** [37]	
33	**HIV1-K03**	CGGGGTTGGGAGGTGGGT		
34	**HIV1-L20**	TGGGAGGGATAAGGGGCGGTTCGGGGA		
35	**HIV1-M27**	GTGGCCTGGGCGGGACTTGGGA		
36	**E-Cote2**	TGGGGAGGGTGGGGAGGGTGGGGAAGG	**Ebola virus** [32]	
37	**E-Cote4**	TGGGATGGGTGGGGTGCTTGTCTGGGGC		
38	**MarRavn**	GTGGTCGGCGTGGGGGGGAGGGT		
39	**c-myc**	TGGGGAGGGTGGGGAGGGTGGGGAAGG	**Others**	
40	**N-myc**	TAGGGCGGGAGGGAGGGAA		
41	**pUC-G1**	GGGGTGTTGGCGGGTGTCGGGGC		
42	**RAN**	TGGGGGTGGGGTTGGGTGGTGT		
43	**RAN-del**	TGGGGGTGGGGTTGGGTGGT		
44	**Z-G4**	TGGTGGTGGTGTGGTGGTGGTGGTGTT		
45	**i-HTR**	CCCAATCCCAATCCCAATCCC	**i-motif**	
46	**i-HTR2**	TCCCAATCCCAATCCCAATCCCA		
47	**C3-Msl1**	CCCTAACCCTAAACCCTAACCC		
48	**AC9**	ACACACACA	**ssDNA**	
49	**AC12**	ACACACACACAC		
50	**AC18**	ACACACACACACACACAC		
51	**AC28**	ACACACACACACACACACACACACACAC		
52	**AT-alt**	ATATATATATATCCCATATATATATAT	**dsDNA**	
53	**GC-alt**	GCGCGCGCGCGCTTTGCGCGCGCGCGC		
54	**ctDNA**	Unspecified calf thymus DNA		
55	**TFO1**	AAAAAAAACCCCTTTTTTTTCCCCTTTTTTTT	**triplex**	
56	**TFO2**	AGAGAGAACCCCTTCTCTCTTATATCTCTCTT		
57	**VK1**	GGGAGCGAGGGAGCG	**AG-tetraplex** [29]	

## Data Availability

The data presented in this study are available in the article and Supplementary Materials.

## Supplementary Materials

The following are available online at https://www.mdpi.com/article/10.3390/ijms22147639/s1, Figure S1: PAGE of DNA oligonucleotides used in this study.

## Abbreviations

CD, circular dichroism; G4, G-quadruplex; G-rich sequence, guanine-rich sequence; ICD, induced circular dichroism; LGGE, ligand gradient gel electrophoresis; mBR, modified Britton-Robinson buffer; RhG, rhodamine 6G; RhB, rhodamine B; TGGE, temperature gradient gel electrophoresis; ThT, thioflavin T; TO, thiazole orange.
