# Supplementary figures and images for: Rhodamine 6G-Ligand Influencing G-Quadruplex Stability and Topology
Source: Int J Mol Sci. 2021 Jul 16;22(14):7639. doi: 10.3390/ijms22147639 (PMC8305571; doi:10.3390/ijms22147639)

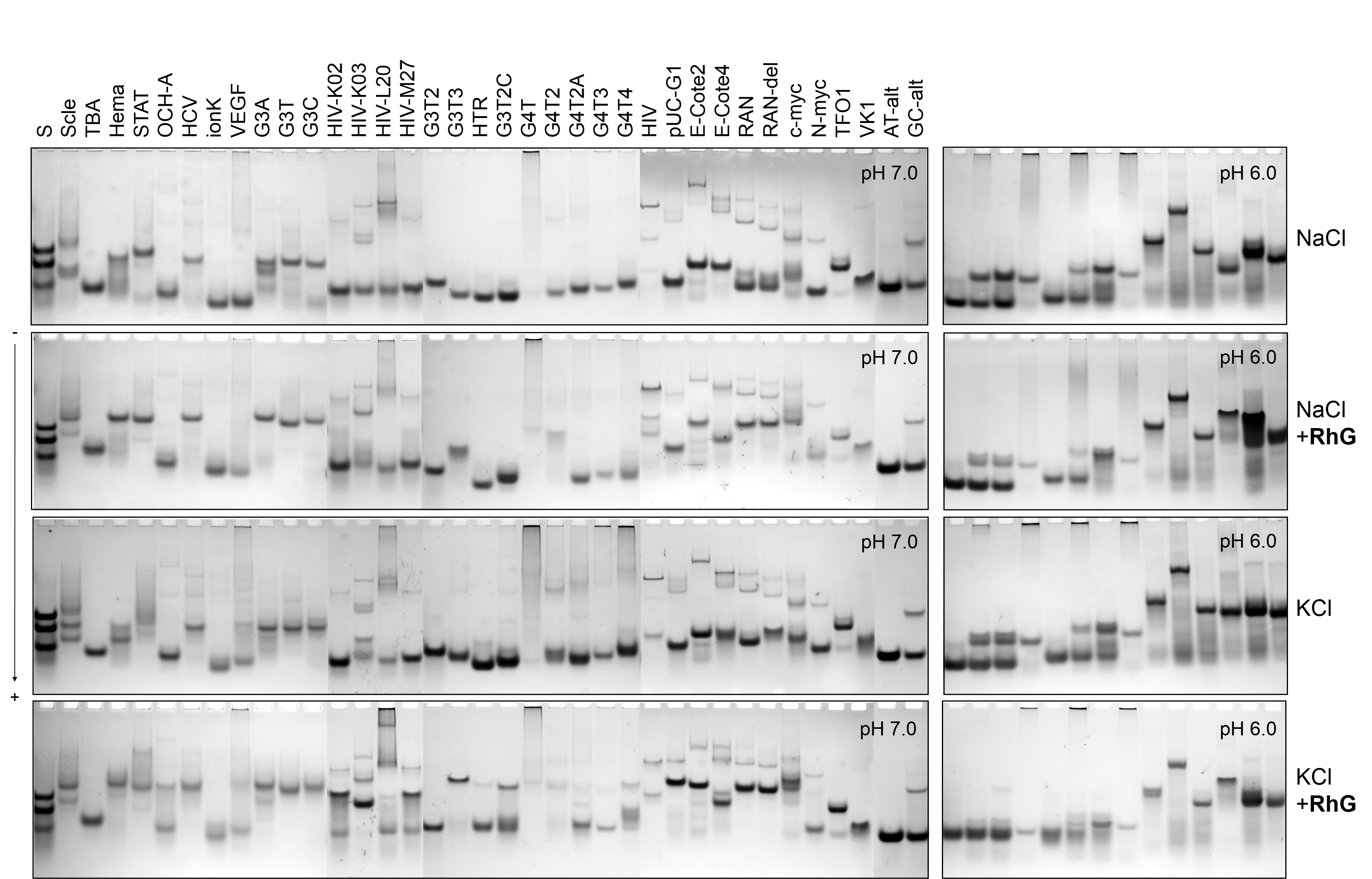

Supplement: Supplementary file 1 [file ijms-22-07639-s001.zip › ijms-1284337-supplementary.tif]
